# Controlling root zone temperature improves plant growth and pigments in hydroponic lettuce

**DOI:** 10.1093/aob/mcad127

**Published:** 2023-09-09

**Authors:** Christopher P Levine, Sota Hayashi, Yoshihiro Ohmori, Miyako Kusano, Makoto Kobayashi, Tomoko Nishizawa, Ikusaburo Kurimoto, Saneyuki Kawabata, Wataru Yamori

**Affiliations:** Institute for Sustainable Agro-ecosystem Services, Graduate School of Agricultural and Life Sciences, The University of Tokyo, Nishitokyo, Tokyo, Japan; Institute for Sustainable Agro-ecosystem Services, Graduate School of Agricultural and Life Sciences, The University of Tokyo, Nishitokyo, Tokyo, Japan; Institute for Sustainable Agro-ecosystem Services, Graduate School of Agricultural and Life Sciences, The University of Tokyo, Nishitokyo, Tokyo, Japan; Faculty of Life and Environmental Sciences, University of Tsukuba, 1-1-1 Tennodai, Tsukuba, Japan; Tsukuba-Plant Innovation Research Center (T-PIRC), University of Tsukuba, 1-1-1 Tennodai, Tsukuba, Japan; Riken Center for Sustainable Resource Science, Yokohama, Kanagawa, Japan; Riken Center for Sustainable Resource Science, Yokohama, Kanagawa, Japan; Riken Center for Sustainable Resource Science, Yokohama, Kanagawa, Japan; Department of Information and Computer Engineering, National Institute of Technology, Kisarazu College, Kisarazu, Chiba, Japan; Institute for Sustainable Agro-ecosystem Services, Graduate School of Agricultural and Life Sciences, The University of Tokyo, Nishitokyo, Tokyo, Japan; Institute for Sustainable Agro-ecosystem Services, Graduate School of Agricultural and Life Sciences, The University of Tokyo, Nishitokyo, Tokyo, Japan

**Keywords:** Root zone temperature, metabolome, pigment, ionome, lettuce (*Lactuca sativa*), plant factory

## Abstract

**Background and Aims:**

Air and root zone temperatures are important environmental factors affecting plant growth and yield. Numerous studies have demonstrated that air temperature strongly affects plant growth and development. Despite the extensive literature on air temperature, comprehensive studies on the effects of root zone temperature (RZT) on plant growth, elemental composition, and pigments are limited. In this study, we carefully observed the effects of RZT in red leaf lettuce to understand its effect on lettuce growth and pigment content.

**Methods:**

Lettuce (*Lactuca sativa*, red leaf cultivar ‘Red Fire’) was grown hydroponically in a plant factory with artificial light under three RZT treatments (15, 25, or 35 °C) for 13 days. We investigated the comprehensive effects of RZT on the production of red leaf lettuce by metabolome and ionome analyses.

**Key Results:**

The 25 °C RZT treatment achieved maximum shoot and root dry weight. The 35 °C RZT decreased plant growth but significantly increased pigment contents (e.g. anthocyanins, carotenoids). In addition, a RZT heating treatment during plant cultivation that changed from 25 to 35 °C RZT for 8 days before harvest significantly increased shoot dry weight compared with the 35 °C RZT and significantly increased pigments compared with the 25 °C RZT. The 15 °C RZT resulted in significantly less pigment content relative to the 35 °C RZT. The 15 °C RZT also resulted in shoot and root dry weights greater than the 35 °C RZT but less than the 25 °C RZT.

**Conclusions:**

This study demonstrated that plant growth and pigments can be enhanced by adjusting RZT during different stages of plant growth to attain enhanced pigment contents while minimizing yield loss. This suggests that controlling RZT could be a viable method to improve lettuce quality via enhancement of pigment content quality while maintaining acceptable yields.

## INTRODUCTION

The world population is estimated to reach 9.7 billion by 2050 (United Nations, Department of Economic and Social Affairs, Population Division, 2019). Meanwhile, conventional farming practices are increasingly unreliable as water availability, a stable climate, and labor availability are all in a state of flux owing to the deleterious effects of climate change and shifting geopolitical landscapes (Benke and Tomkins, 2017; Al-Kodmany, 2018; Kwon *et al.*, 2019). Controlled environment agriculture (CEA) is one way of food production that can potentially increase the reliability of a stable domestic fresh produce supply chain as these three aforementioned variables increasingly disrupt society (Kozai, 2013; Merrill *et al.*, 2016). However, CEA has significant barriers that restrict its ability to upscale meaningfully and contribute to the global food supply chain (Engler and Krarti, 2021). Farm system optimization, from ‘fundamental plant physiology and applied physics to social science’, has been recommended (Van Delden *et al.*, 2021).

Multiple studies have examined the effects of air temperature on plant growth over a substantial range (Way and Yamori, 2014; Yamori *et al.*, 2014). In general, low or high air temperatures negatively affect plant physiological processes such as photosynthesis, respiration, growth, and development, resulting in reduced crop yields (Hasanuzzaman *et al.*, 2013). The root zone temperature (RZT) has also been reported to be important for plant growth and development because it affects root physiological processes such as water and nutrient uptake. Studies have demonstrated that RZT affects photosynthesis and assimilate partitioning (Ruter *et al.*, 1992). Moreover, increasing the RZT in tomatoes created differences in leaf carbon exchange rate and parallel changes in carboxylase activity induced by warming the roots at ambient temperature (Hurewitz and Janes, 1987). Studies have also demonstrated that the RZT affects water and mineral nutrient uptake (Udagawa *et al.*, 1991; Klock *et al.*, 1996;Mackay and Barber, 1984; Quadir *et al.*, 2011). In particular, for strawberry growth, the water absorption rate was hastened at the beginning of treatment and subsequently decreased, in addition to reduced nutrient uptake during the final 20 days of treatment (Udagawa *et al.*, 1991). This observation suggests increased plant stress responses from supra-optimal RZTs. Furthermore, prior research has demonstrated that the application of water artificially heated to 23 °C to eight herbaceous plant species caused them to die faster or at higher proportions and to have minor reductions in growth and biomass relative to the colder, 18 °C water temperature treatment (Main *et al.*, 2022). Overall, these studies suggest that RZT should be a considered factor, especially when growing plants in a hydroponic plant factory environment (Poorter *et al.*, 2012). When the RZT is low or high, plants activate antioxidant reactions (Li *et al.*, 2019). Also, the optimal RZT varies with air temperature (Yamori *et al.*, 2022). Root growth increases linearly with increasing temperature from the minimum to the optimal temperature, but further increases in RZT are accompanied by a rapid decrease in root and shoot growth (Arkin and Taylor, 1981; David and Arnison, 1986; Yamori *et al.*, 2022). Other studies even suggest that RZT appears to be more critical than air temperature in controlling plant growth (Aldous and Kaufmann, 1979; Kuroyanagi and Paulsen, 1988; Paulsen, 1994; Xu *et al.*, 2001). Controlling the RZT increases the resource-use efficiency in red-leaf lettuce, especially if the water source is naturally derived from a low-temperature (<15 °C) or high-temperature (>35 °C) source. Furthermore, depending on the time of the year (i.e. winter or summer), the temperature of the water source can vary significantly. How the water is stored at the CEA operation must also be considered (e.g. insulated tanks outside or non-insulated tanks in a climate-controlled room).

Separate control over the RZT and air temperature affects CEA operations from a plant physiological viewpoint, given that previous studies have suggested that the RZT applied to various species has a strong influence on plant growth and that RZT and air temperature can lead to different physiological responses (Sattelmacher *et al.*, 1990; Pardales *et al.*, 1991; Ruter and Ingram, 1992; McMichael and Burke, 1994; Udomprasert *et al.*, 1995; Ziska and Bunce, 1998; Xu *et al.*, 2001; Yamori *et al.*, 2022). Resource-use efficiency is crucial in CEA because the yield benefits need to justify the increased production environment costs relative to conventional outdoor field agriculture (Lubna *et al.*, 2022). Currently, many CEA operations globally ignore RZT control even though it has significant plant physiological responses and potential economic benefits for CEA operations. High and low RZTs are known to create various physiological responses in plants (Li *et al.*, 2019; Yamori *et al.*, 2022). However, the effects of these temperature changes on red leaf lettuce physiological processes and the underlying mechanisms have not yet been analyzed in detail. In this study, we intended to clarify how changes in RZT affect the production of hydroponically grown red leaf lettuce in controlled environments. We investigated the comprehensive effects of RZT on the production of red leaf lettuce by metabolome and ionome analyses. Ultimately, this research could provide CEA operations with more insight into how implementation of dynamic RZT control can amplify certain desirable crop characteristics, which include but are not limited to specific pigments, shoot mass and leaf pigments. Optimizing RZT is not the sole answer to address the food needs of an increasing world population. Instead, optimizing RZT is an additional tool that can be used for a healthier diet. Increasing pigment content to increase the perception of quality could possibly increase the consumption of the vegetables necessary for healthy diets.

The application of abiotic stressors to amplify stress responses in plant factories has been suggested to increase the consumer’s initial perception of product quality (Ryder, 1999; Gazula *et al.*, 2007; Lightbourn *et al.*, 2008). The cultivation of plants is usually managed in a way that avoids environmental stress responses. However, applying environmental stress to plants to the extent that it does not cause extreme growth inhibition or irreversible stress disorders could result in certain benefits.

## MATERIALS AND METHODS

### Plant material and treatments

The experiment was conducted in a plant factory with artificial light, located at the Graduate School of Agricultural and Life Science, Institute for Sustainable Agro-ecosystem Services, The University of Tokyo (Nishi-Tokyo City, Tokyo: 35°43ʹN, 139°32ʹE). The seeds of leaf lettuce (*Lactuca sativa*, cultivar red leaf ‘Red Fire’, Takii Seed Co., Kyoto, Japan) were used. Growth conditions were controlled at an air temperature of 25/22 ± 1 °C (day/night), relative humidity of 62.9 ± 6 %, and 16 h photoperiod. During the seed propagation period, the light intensity was maintained at 120 ± 10 µmol m^−2^ s^−1^ photosynthetic photon flux density (PPFD) during the daytime. After the lettuce plants were transplanted, the light intensity was increased to a PPFD of 200 ± 20 µmol m^−2^ s^−1^, using white LEDs (TecoG II-40N2-5-23, Toshin Electric Co., Ltd, Osaka, Japan).

The seed sowing to planting lasted 13 days. After 13 days, the plants were acclimated to the experiment system set-up without RZT control for 3 days. Following the acclimation period, the RZT treatment to harvest trial took place over 16 days. The plants were grown in a custom-built nutrient film technique system, supplied with a hydroponic nutrient solution (electrical conductivity: 1.00 ± 0.05 dS m^−1^; GG liquid A & B stock solutions, Green Green Co., Ltd, Fukuoka, Japan).

Experiment one involved three RZT treatments: 15, 25, and 35 °C. The RZT was controlled with either a heater (NHA-065, Marukan Co., Ltd, Osaka, Japan) or a cooler (ZR mini, Zensui Co., Ltd, Osaka, Japan), which was installed in the reservoir to keep a consistently stable nutrient solution temperature over the entire duration of the experimental run. The three RZTs (15, 25, and 35 °C) were selected for three reasons. First, we selected 25 °C RZT to be in line with the air temperature used for commercial production. Second, the 15 and 35 °C RZTs were selected based on our prediction that these large differences might lead to stress responses. Third, we wanted to provide consistently different RZTs, and selecting smaller differences in RZT from the air temperature might not have enabled consistent RZT differences over the entire treatment duration. A sensor (TR-52i, T&D Holdings, Inc., Tokyo, Japan) was used to monitor RZT throughout the experiment to ensure that significant deviations were avoided. Dissolved oxygen was measured using a hand-held meter (LAQUA D-200, Horiba, Ltd, Kyoto City, Japan).

For the second experiment, in which the RZT was altered during the cultivation process, the same growing method, protocols, and systems were used as stated for experiment one. For example, similar to experiment one, the sowing to planting occurred over 13 days, acclimation to the experimental system set-up was 3 days, and RZT treatment to harvest was 16 days. The leaves used for metabolite analysis were harvested 16 days after the experimental trial start time. The only two differences between experiment one and experiment two were that for experiment two, the plants were grown in a total of five RZT treatments: 25 °C; HT1 (12 days of 25 °C followed by 4 days of 35 °C); HT2 (8 days of 25 °C followed by 8 days of 35 °C); HT3 (4 days of 25 °C followed by 8 days of 35 °C); and 35 °C treatments. The other difference was that plants were grown under a consistent air temperature of 22 °C during the day and night over an experiment trial time of 16 days after acclimation rather than adjusting day and night air temperatures as in experiment one.

### Plant growth and photosynthesis

Seventy-two plants were randomly selected and divided into three treatments. Of the 24 plants in each treatment, six to nine were collected for shoot and root dry mass, dry shoot-to-root ratio, and root length and volume parameters. Seven to ten of the 24 plants per treatment were collected for pigment analysis, and six plants were collected for leaf and root metabolite analysis. For dry weight, the shoots and roots from every plant were separated, placed in paper envelopes, and then dried at 80 °C in a constant-temperature oven for ~2 weeks. For analysis of root characteristics, shoots and roots were separated with tweezers immediately after harvest, placed in water-filled trays to minimize root overlap, and scanned. The scanned images were analyzed using WinRHIZO software (Regent Instruments Inc., Québec City, QC, Canada, 2000) to calculate the values for total root length and root volume.

The quantum yields of photosystem II [Y(II)] were obtained as described previously (Klughammer and Schreiber, 1994; Baker, 2008) in each of the growth conditions using plants immediately before sample collections for plant growth analysis. The electron transport rates around photosystems I and II (ETR I and II) were calculated using the following equation: ETR II = 0.5 × abs I × Y(II), where 0.5 is the fraction of absorbed light allocated to photosystem I or II, and abs I refers to leaf absorbance, taken as 0.84 of the incident irradiance (i.e. 200 µmol m^−2^ s^−1^).

### Pigments

The contents of chlorophyll, carotenoid, and anthocyanin were measured for the pigment analysis. Two 0.56 mm^2^ holes were punched in the center of the largest fully expanded leaf blades. One millilitre of 80 % acetone was added to the cut sample and ground with a mortar and pestle to extract chlorophyll and carotenoids. The acetone extract was centrifuged at a g-force of about 8855 g for 5 min, and the supernatant was used for the analysis. Analysis was performed by measuring absorbance at 750.0, 636.6, 646.6 and 470.0 nm using a UV-Vis-NIR spectrophotometer (UV-2700, Shimadzu Corporation, Kyoto, Japan), and the values of chlorophyll and carotenoid content were determined using equations derived from Porra *et al.* (1989) and Lichtenthaler (1987). Anthocyanin content was measured in the leaf blade of the largest leaf using an anthocyanin content meter (ACM-200 plus, Einex Corporation, Tokyo, Japan).

### Ionome analysis

Leaf and root samples were dried for 3 days in an oven at 70 °C. After the samples had been dried and ground, 40–50 mg per sample were collected for further analysis. Each sample was digested with concentrated nitric acid (NHO_3_) and hydrogen peroxide (H_2_O_2_) (FUJIFILM Wako Pure Chemical Corporation, Osaka, Japan) as follows: 30 min at 80 °C and 1 h at 120 °C with 2 mL NHO_3_, 1 h at 120 °C after adding 0.5 mL NHO_3_ and 0.5 mL H_2_O_2_, and overnight at 80 °C until the samples were completely dried. The dried pellets, after digestion were dissolved in 1 mL of 0.08 m HNO_3_ for the inductively coupled plasma mass spectrometry (ICP-MS) sample. This digestion with HNO_3_ is similar to elemental analysis digestion conducted in an independent commercial testing laboratory for absolute values, as described by Levine and Mattson (2021). Elemental concentrations in 10-fold diluted ICP-MS samples [phosphorus (P), potassium (K), calcium (Ca), magnesium (Mg), sulfur (S), iron (Fe), manganese (Mn), boron (B), zinc (Zn), molybdenum (Mo), copper (Cu), nickel (Ni), sodium (Na), cobalt (Co), lithium (Li), germanium (Ge), arsenic (As), selenium (Se), rubidium (Rb), strontium (Sr), cadmium (Cd) and caesium (Cs)] were measured by ICP-MS (Agilent 7800, Agilent Technologies Co., Ltd, Japan) using 2 ppb indium as an internal standard, as described by Monden *et al.* (2022). Next, the plant concentration (in parts per billion) of each element was calculated and compared among treatments as relative values. Six biological replicates were used for the analysis.

### Metabolite profiling analysis

We conducted metabolite profiling using gas chromatography–time-of-flight mass spectrometry as described by Kusano *et al.* (2007a,b), with slight modifications. Six biological replicates were used for the analysis. Samples were collected and frozen for analysis based on methods described by Kitazaki *et al.* (2018). Metabolites were extracted from each leaf and root sample at a concentration of 2.5 mg dry weight of tissue per millilitre of extraction solution (methanol:chloroform:water = 3:1:1 v/v/v). The extracted samples were methoxylated and subsequently trimethylsilylated. An equivalent of 5.6 µg dry weight of derivatized samples was subjected to gas chromatography–time-of-flight mass spectrometry. Obtained data network common data (NetCDF) were transferred to MATLAB software v.2011b (MathWorks, Natick, MA, USA). The chromatograms were preprocessed using the high-throughput data analysis method (Johnson *et al.*, 2005) and normalized using the cross-contribution compensating multiple standard normalization algorithm (Redestig *et al.*, 2009).

### Statistical analysis

Significant difference tests for the means of measurement values were performed by a Tukey–Kramer honestly significant difference (HSD) test at α = 0.05 to determine significant differences among measured parameters.

For elemental and metabolite content, significance difference tests of means in comparison to the 25 °C treatment (the control) were performed with the drc 3.0-1 package (Ritz *et al.*, 2015) of the software R v.3.6.2 (R Core Team, 2017), using the graphical interface RStudio Desktop v.1.1.4.6.3 (RStudio Team, 2015).

## RESULTS

### Root zone temperature affects plant growth and pigments

**Fig. 1.**
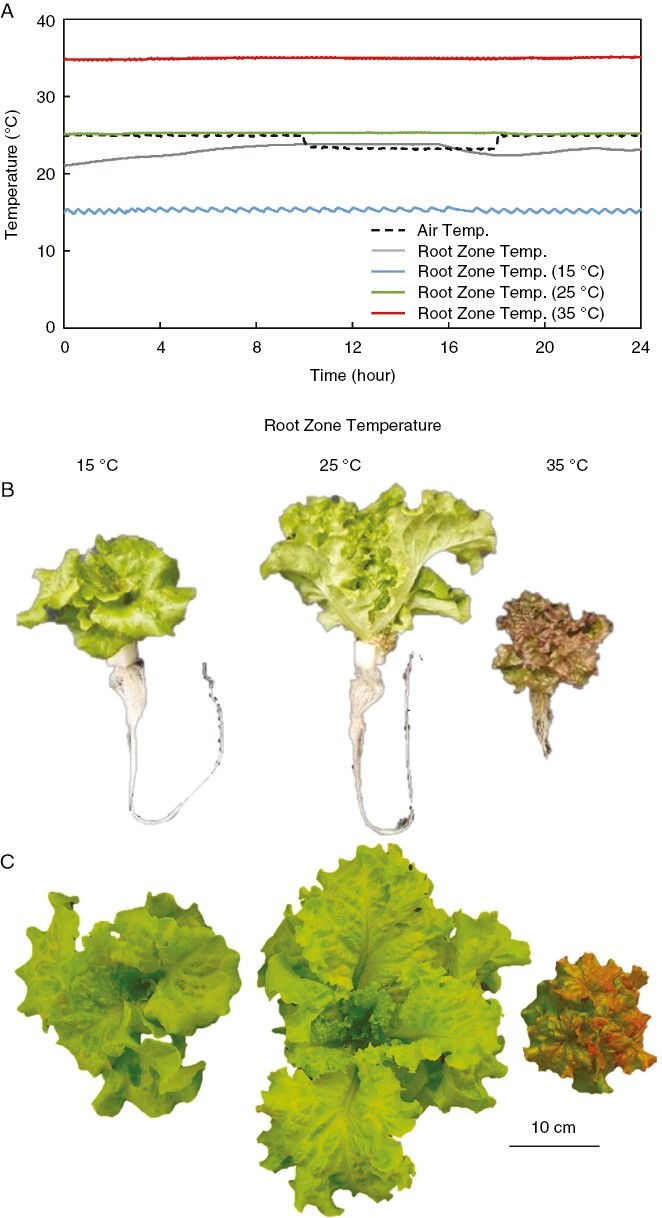
(A) Time course of air temperature and nutrient solution temperature when root zone temperature is controlled at 15, 25 or 35 °C. (B, C) Side view (B) and top view (C) of red leaf lettuce harvested 32 days after sowing in 15, 25, and 35 °C treatments.

**Fig. 2.**
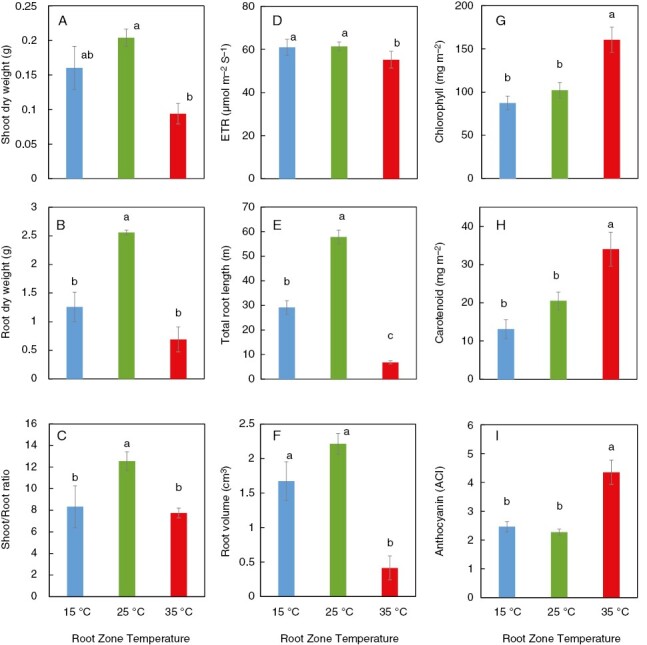
Shoot dry weight (A), root dry weight (B), shoot-to-root ratio (C), photosynthetic electron transport rate (ETR; D), total root length (E), root volume (F), chlorophyll content (G), carotenoid content (H) and anthocyanin content (I) of red leaf lettuce at a root zone temperature (RZT) of 15, 25 and 35 °C. Bars are standard errors (*n* = 6–10). Lower case letters indicate significant differences by Tukey’s HSD test (5 % level of significance).

Plants were grown at different constant RZTs of 15, 25, and 35 °C under an air temperature of 22 °C (Fig. 1A). In comparison to the 25 °C RZT treatment, the root dry weight, shoot-to-root ratio, total root length and root volume were all significantly decreased at both 15 and 35 °C, and shoot dry weight significantly decreased at 35 °C (Figs 1B, C and 2A–E). The content of chlorophyll, carotenoids, and anthocyanins in leaves increased significantly in the 35 °C treatment compared with the 15 and 25 °C treatments (Fig. 2F–H).

### Root zone temperature affects the elemental concentration and metabolic profiles of the plant

**Fig. 3.**
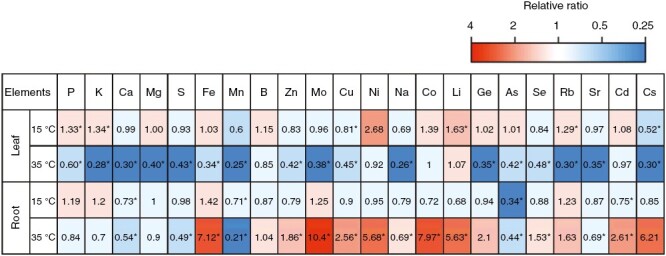
Relative ratio in the amount of each element in the leaves and roots of red leaf lettuce grown at a root zone temperature (RZT) of 15 and 35 °C compared with the 25 °C treatment. *Significant differences by Student’s *t*-test (5 % level of significance).

Root zone temperature greatly affected the elemental concentration of both the leaves and the roots (Fig. 3). In comparison to the 25 °C treatment, plants in the 15 °C treatment experienced a significant decrease in Ca, Mn, As, and Cd in the roots, with significant increases of P, K, Li, and Rb and significant decreases of Cu and Cs in leaves (Fig. 3). In comparison to the 25 °C treatment, the 35 °C treatment led to significant increases in Fe, Zn, Mo, Cu, Ni, Co, Li, Se and Cd and significant decreases of Ca, S, Mn, As and Sr in roots. Concurrently, with the 35 °C treatment, significant decreases of P, K, Ca, Mg, S, Fe, Mn, Zn, Mo, Cu, Na, Ge, As, Se, Rb, Sr, and Cs occurred in the leaves (Fig. 3).

**Fig. 4.**
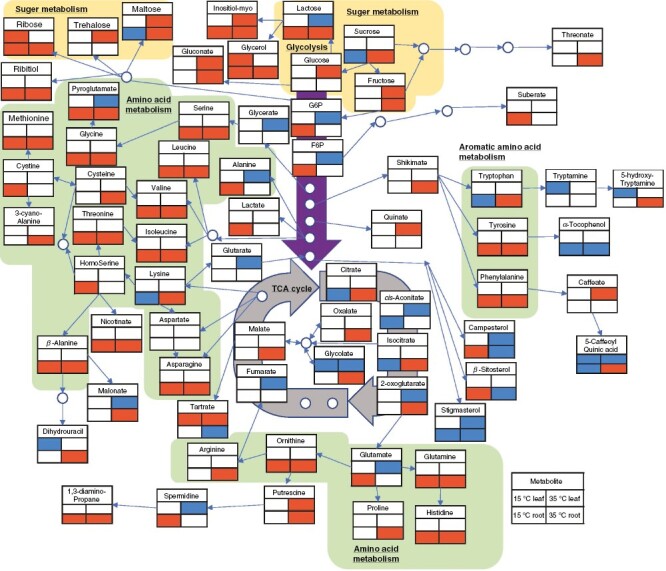
Changes in the amount of each metabolite in the leaves and roots of red leaf lettuce grown at a root zone temperature of 15 and 35° C compared with the 25 °C treatment. The lower left box of the metabolite names represents changes in metabolites in the leaves and the lower right represents changes in metabolites in the roots. Significant increases by Student’s *t*-test (5 % level of significance) are shown in red and decreases in blue.

The RZT also significantly affected plant metabolite profiles in both the leaves and the roots (Fig. 4). In comparison to the 25 °C treatment, the concentrations of 19 amino acids (isoleucine, serine, tyrosine, valine, methionine, leucine, cystine, phenylalanine, threonine, homoserine, histidine, pyroglutamate, alanine, glutamate, glutamine, ornithine, glycine, β-alanine, and asparagine) in the 15 °C treatment were significantly increased in the roots. At the same time, the leaf profiles were not significantly changed (Fig. 4). In contrast, in comparison to the 25 °C treatment, the 35 °C treatment significantly affected the metabolite profiles in both the roots and leaves. In roots, there were significant increases of seven sugars (glucose, fructose, sucrose, turanose, lactose, ribose, and maltose), 21 amino acids (arginine, phenylalanine, lysine, glycine, histidine, proline, cysteine, isoleucine, threonine, tyrosine, glutamine, valine, asparagine, pyroglutamate, β-alanine, 3-cyano-alanine, leucine, tryptophan, ornithine, serine, and methionine) and five metabolites in the tricarboxylic acid (TCA) cycle (isocitric acid, 2-oxo-glutaric acid, citric acid, fumaric acid, and malic acid). The concentrations of five sugars (glucose, fructose, turanose, trehalose, and maltose) were increased in the leaf tissue. Four metabolites in the TCA cycle (malic acid, fumaric acid, 2-oxo-glutaric acid, and *cis*-aconitic acid) and two metabolites in the glycolytic system [glucose-6-phosphate (G6P), and fructose-6-phosphate (F6P)] were decreased (Fig. 4). Figs 3 and 4 broadly show that elements and metabolites are important for crop growth and food. Our results show that rhizosphere temperature broadly alters the above- and below-ground accumulation of numerous elements and metabolites. When RZTs are adjusted, numerous complex chemical reactions occur, all affected in one way or another.

### Clustering of elements and metabolites altered by RZT treatment

Network analysis by weighted correlation network analysis was used to cluster metabolites and elements that were altered by the RZT treatment (Figs 3 and 4). The clustering classified those metabolites and elements into multiple groups. Enrichment analysis (Metabo v.4.0) was also used to determine the metabolic pathways of these groups enriched for metabolites.

First, metabolites and elements that were significantly changed in the roots by the 15 °C RZT compared with the 25 °C RZT could be divided into three groups. Group 1 contained 19 metabolites and one element, group 2 contained eight metabolites, and group 3 contained 14 metabolites and 20 elements (Supplementary Data Fig. 1A, B). Group 1 was significantly enriched with valine, leucine, and isoleucine biosynthesis, glutamine, and glutamate metabolic pathways, neomycin, kanamycin, and gentamicin biosynthesis (Supplementary Data Fig. 1C). At the same time, group 2 contained phenylalanine, tyrosine, and tryptophan biosynthesis and d-glutamine and d-glutamate metabolism (Supplementary Data Fig. 1D), and group 3 was significantly enriched for galactose metabolism (Supplementary Data Fig. 1E). Group 1 was not significantly correlated with plant growth or pigments (Supplementary Data Fig. 1A). In contrast, groups 2 and 3 were negatively correlated with shoot dry weight, root dry weight, total root length and root volume and positively correlated with chlorophyll, carotenoids, and anthocyanins (Supplementary Data Fig. 1A).

Second, metabolites and elements in leaves significantly altered by RZT treatment at 15 °C compared with 25 °C could be divided into two groups. Group 1 contained five metabolites and seven elements, while group 2 contained ten metabolites and three elements (Supplementary Data Fig. 2B). The metabolites and metabolic pathways of the groups did not exhibit significant enrichment signs (Supplementary Data Fig. 2C, D). Groups 1 and 2 were positively correlated with shoot dry weight, root dry weight, total root length and root volume and negatively correlated with chlorophyll, carotenoids and anthocyanins (Supplementary Data Fig. 2A).

**Fig. 5.**
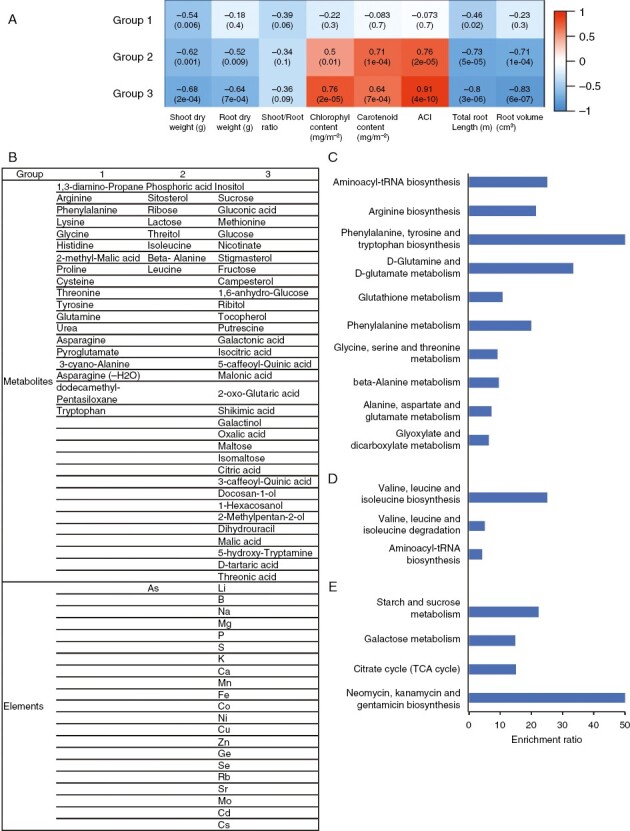
Network analysis by weighted correlation network analysis was used to cluster metabolites and elements in roots that were altered by the root zone temperature treatment. The clustering was classified into three groups. (A) The link between each group (1, 2 and 3) and its correlation with each respective phenotype. (B) Groups of measured elements and metabolites with significant differences (*P* < 0.05 by Student’s *t*-test) in roots in the 25 and 35 °C treatments. (C) Metabolite enrichment analysis of group 1. (D) Metabolite enrichment analysis of group 2. (E) Metabolite enrichment analysis of group 3.

Third, the metabolites and elements that were significantly altered in the roots by RZT at 35 °C compared with 25 °C could be divided into three groups. Group 1 contained 19 metabolites, group 2 contained eight metabolites, and group 3 contained 33 metabolites and 21 elements (Fig. 5A, B). Group 1 was significantly enriched in phenylalanine, tyrosine, and tryptophan biosynthesis and in d-glutamine and d-glutamate metabolism (Fig. 5C), while group 2 was significantly enriched in valine, leucine, and isoleucine biosynthesis (Fig. 5D), and group 3 was significantly enriched in starch and sucrose metabolism, galactose metabolism, citrate cycle (TCA cycle), neomycin, kanamycin and gentamicin biosynthesis (Fig. 5E). Group 1 showed no significant correlation with plant growth or pigments. In contrast, groups 2 and 3 were negatively correlated with shoot dry weight, root dry weight, total root length, and root volume and positively correlated with chlorophyll, carotenoids, and anthocyanins (Fig. 5A).

**Fig. 6.**
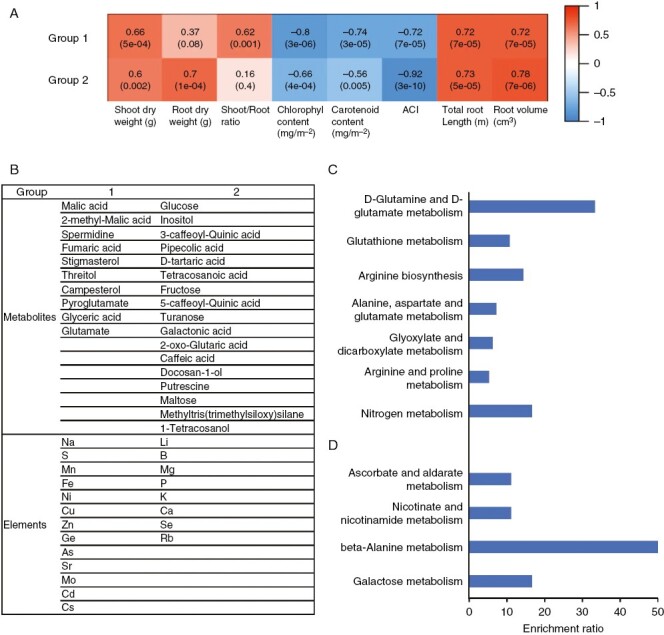
Network analysis by weighted correlation network analysis was used to cluster metabolites and elements in leaves that were altered by the root zone temperature treatment. The clustering was classified into three groups. (A) The link between each group (1 and 2) and its correlation with each respective phenotype. (B) Groups of measured elements and metabolites with significant differences (*P* < 0.05 by Student’s *t*-test) in leaves in the 25 and 35 °C treatments. (C) Metabolite enrichment analysis of group 1. (D) Metabolite enrichment analysis of group 2.

Fourth, metabolites and elements in leaves significantly altered by the 35 °C RZT treatment compared with 25 °C RZT could be divided into two groups. Group 1 contained ten metabolites and 13 elements, while group 2 contained 17 metabolites and eight elements (Fig. 6B). Group 1 was significantly enriched in d-glutamine and d-glutamate metabolism and nitrogen metabolism (Fig. 6C), while group 2 was significantly enriched in β-alanine metabolism and galactose metabolism (Fig. 6D). Group 1 and 2 were positively correlated with shoot dry weight, root dry weight, total root length and root volume and negatively correlated with chlorophyll, carotenoids and anthocyanins (Fig. 6A).

### Altering RZT during the cultivation process affects plant growth and pigment content

**Fig. 7.**
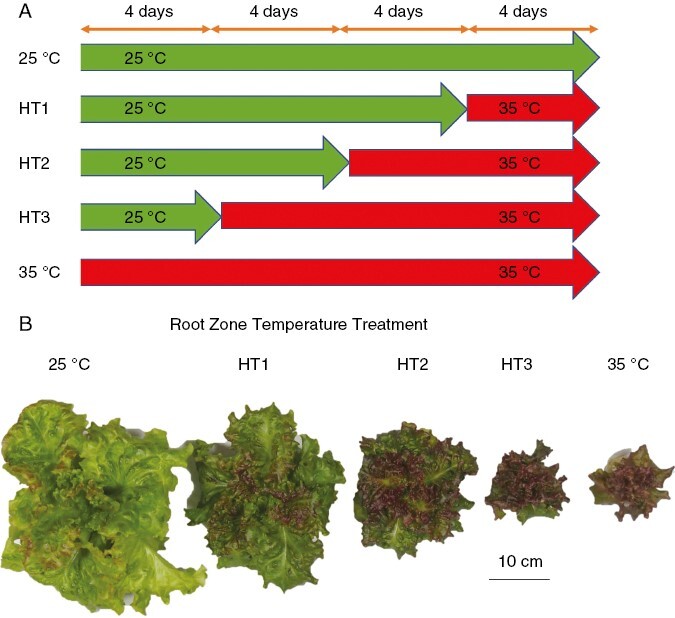
An experiment in which the root zone temperature (RZT) was altered to a high temperature (HT) during plant cultivation. (A) Cultivation treatments for HT1, HT2 and HT3. (B) Top view of red leaf lettuce from 25 °C, HT1, HT2, HT3, and 35 °C treatments harvested 32 days after sowing. Plants were grown in a total of five RZT treatments: 25 °C; HT1 (12 days of 25 °C followed by 4 days of 35 °C); HT2 (8 days of 25 °C followed by 8 days of 35 °C); HT3 (4 days of 25 °C followed by 8 days of 35 °C); and 35 °C treatments under 22 °C air temperatures 16 days after sowing.

**Fig. 8.**
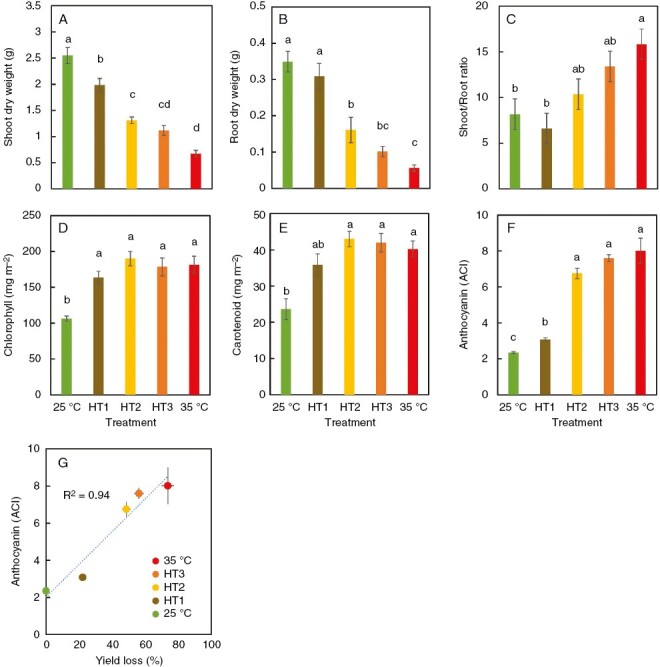
An experiment in which the root zone temperature was altered to a high temperature (HT) during plant cultivation. (A–F) Shoot dry weight (A), root dry weight (B), shoot-to-root ratio (C), chlorophyll (D), carotenoid (E) and anthocyanin (F) contents of red leaf lettuce in 25 °C, HT1, HT2, HT3 and 35 °C treatments. (G) Relationship between yield loss and anthocyanin content. Bars are standard errors (*n* = 5–10). Significant differences between different letters by Tukey’s HSD test (5 % level of significance).

Given that the high RZT treatment contributed to the improvement of pigment contents but negatively affected plant growth (Figs 1–4), we conducted a second experiment, in which the RZT was altered to a high temperature (HT) during the growing process. Plants were grown in a total of five RZT treatments: 25 °C; HT1 (12 days of 25 °C followed by 4 days of 35 °C); HT2 (8 days of 25 °C followed by 8 days of 35 °C); HT3 (4 days of 25 °C followed by 8 days of 35 °C); and 35 °C under 22 °C air temperatures (Fig. 7). The increased duration of the 35 °C RZT treatment resulted in a reduction in shoot dry weight and root dry weight and increases of the chlorophyll, carotenoid and anthocyanin contents (Fig. 8). Also, chlorophyll content increased significantly with 35 °C RZT treatment for >4 days, while carotenoid and anthocyanin contents increased significantly with 35 °C RZT treatment for >8 days (Fig. 8).

## DISCUSSION

Hydroponic lettuce production in CEA has been widely practiced, and various studies have been conducted to control the environment for optimal plant growth. RZT has been considered to affect plants more than the air temperature, but the approximate RZT thresholds to enable quality lettuce production in CEA have not been established. Therefore, it is important to clarify the physiological processes by which RZT affects plant growth in response to different RZT controls in order to establish the optimal control of RZT for lettuce production.

First, we found that 15 °C compared with 25 °C RZT treatment reduced plant growth owing to significantly reduced root growth and partly reduced shoot growth (Figs 2 and 3) This reduction might be attributable to reduced photosynthesis and water uptake, but this is difficult to conclude because these parameters were not measured. The electron transport rate for photosystem II (ETR II) indicated that the plants with 35 °C RZT experienced significantly reduced ETR II values relative to the 15 and 25 °C RZTs.

Second, we found that 35 °C compared with the 25 °C RZT treatment, restricted plant growth in roots and leaves owing to the reduced root growth but enhanced pigments including anthocyanin, chlorophyll, and carotenoids (Fig. 2). This was probably attributable to a typical heat stress response.

Third, we found that the longer the period of 35 °C RZT treatment, the more the contents of anthocyanin, carotenoids, and chlorophyll increased while shoot dry weight decreased, indicating that 35 °C RZT treatment for 4 days before harvest improves pigments while minimizing the reduction in plant growth compared with a consistent 35 °C RZT throughout the entire cultivation period (Figs 7 and 8). In summary, low and high RZT temperatures limit plant growth through different mechanisms, and high RZT temperature treatment before harvest could somewhat reduce production but accumulate pigment contents, leading to increased value-added lettuce.

### Lettuce response to low RZT

The 15 °C RZT treatment reduced root growth, suggesting that this might have led to reduced leaf growth (Fig. 2). Accumulation of several amino acids (isoleucine, serine, tyrosine, valine, methionine, leucine, cystine, phenylalanine, threonine, homoserine, histidine, pyroglutamate, alanine, glutamate, glutamine, ornithine, glycine, β-alanine, and asparagine) was observed in roots at 15 °C RZT (Fig. 4). In the roots of cold-tolerant rice cultivars, differentially expressed genes related to amino acid metabolism, such as α-aminoadipic semialdehyde synthase, tyrosine aminotransferase, and arginine decarboxylase, might have been responsible for the increase in most amino acids under cold stress (Fang *et al.*, 2021). These differentially expressed genes related to amino acid metabolism might have influenced the increase in amino acid content at 15 °C RZT, and thus, in the present study, 15 °C RZT inhibited root growth. Accumulation of several amino acids at 15 °C RZT could be attributable to the progressive degradation or retardation of proteins, because, in plants subjected to low temperature treatments, more energy carriers are consumed to enhance adaptation to low temperatures, producing lipids, amino acids, and other molecules, in addition to promoting cell membrane fluidity and structural rearrangement (Maruyama *et al.*, 2014; Wu *et al.*, 2016). The degradation or retardation of proteins owing to amino acid build-up are mere predictions, and other factors could possibly be involved. Another possibility is that cold temperatures also caused the destabilization of protein complexes (Thakur and Nayyar, 2013).

Also, the 15 °C RZT seemed to affect d-glutamine and d-glutamate metabolism and galactose metabolism in the roots (Supplemental Data Fig. 2). These metabolic changes might affect amino acid accumulation. Furthermore, an increase in G6P and F6P was observed in roots at 15 °C RZT (Fig. 4). It is known that the expression of fructokinase is increased in rice roots under cold stress (Lee *et al.*, 2009). Therefore, this increased expression of fructokinase might explain the increase in G6P and F6P in roots at 15 °C RZT, and the increase in G6P and F6P in roots in the 15 °C RZT treatment suggests that the metabolism would have been stalled by the low temperature.

In contrast, the content of pigments in leaves did not change, but shoot dry weight seemed to decrease (Fig. 2). Given that groups containing the most elements at 15 °C RZT in roots were negatively correlated with plant growth (Supplementary Data Fig. 1), the reduction in shoot dry weight might have been caused by reduced uptake of elements by the roots owing to reduced root growth. It has been suggested that hydroponic plant production at an RZT of 10 °C induces an oxidative stress response and activates antioxidant secondary metabolism in lettuce leaves (Sakamoto and Suzuki, 2015). However, in our experiments, the antioxidant effects of anthocyanins and carotenoids were not significantly increased in leaves at 15 °C RZT, because there was no increase in antioxidant metabolites or compatible solutes in the leaves at an RZT of 15 °C (Fig. 4). Thus, lower RZT treatment below 15 °C could be necessary to induce an oxidative stress response and thus induce accumulation of antioxidant metabolites in leaves. However, if the protein metabolism is greatly affected, it is also expected to be more susceptible to oxidative stress.

### Lettuce response to high RZT

The 35 °C RZT treatment reduced root growth, hence plant growth (Fig. 2).

First, in comparison to the 25 °C RZT, the 35 °C RZT treatment increased the concentration of many elements in the roots and decreased most elements in the leaves (Fig. 3). Previous studies have indicated that high RZT inhibits the transport of nutrients and water from roots to leaves (Graves *et al.*, 1991; Klock *et al.*, 1997; Huang, 2000). In fact, groups containing few elements in the 35° C RZT treatment showed a negative correlation with plant growth (Fig. 5). Thus, the 35 °C RZT might have inhibited the transport of elements from the roots to the leaves, consequently resulting in the accumulation of many elements in the roots.

Second, the 35 °C RZT increased amino acids, such as proline, sugars, such as sucrose, and metabolites in the TCA cycle in the roots (Fig. 4). Given that it is well known that proline concentration increases in various stressful conditions (Bray, 1993;Kusano *et al.*, 2011), the increase in proline concentration observed in the present study indicates that 35 °C RZT caused a stress response in the roots. This observation is supported by the fact that the 35 °C RZT group was enriched in the roots with valine, leucine, and isoleucine biosynthesis, which are generally considered to increase with environmental stress (Fig. 5). In addition, it has been reported that, under high RZT stress, root proteins decreased while sucrose metabolism was activated to store energy for root survival, resulting in the accumulation of sugars (Du and Tachibana, 1994; Huang *et al.*, 2012). Therefore, in roots at 35 °C RZT, amino acids might have accumulated without progressing to protein formation, and sugars might have accumulated owing to the activation of sucrose metabolism (Fig. 4). In fact, the 35 °C group is enriched in the roots with starch and sucrose metabolism and galactose metabolism (Fig. 5). At the high RZT, the rate of cytochrome respiration was decreased, but the rate of alternative respiration was increased, resulting in increases in total root respiration in mitochondria (Du *et al.*, 1994). Therefore, it is possible that the 35 °C RZT treatment activated the TCA cycle to produce secondary metabolites and to release the excess energy produced to the alternative pathway (Fig. 4). The group at 35 °C RZT was enriched in the roots with the TCA cycle (Fig. 5).

Third, it should not be ignored that higher RZT decreases the concentration of dissolved oxygen in the nutrient solution (Supplementary Fig. 3), which might cause root browning and negatively affect root growth (Chun and Takakura, 1994; Falah *et al*., 2010).

Pigments, including anthocyanins, carotenoids and chlorophyll, in leaves were significantly increased in the 35 °C RZT, although it reduced plant growth (Fig. 2). Plants normally accumulate reactive oxygen species in stress conditions, and plant tolerance to stress is often associated with increased antioxidant enzyme activity (Sairam and Saxena, 2000; Ding *et al.*, 2016). Therefore, the increase in anthocyanins and carotenoids in leaves at 35 °C RZT could be attributed to the generation of reactive oxygen species in leaves owing to high-temperature stress responses and the increase in antioxidants for their removal. Carotenoids are produced from β-alanine via acetyl CoA, but the 35 °C group was enriched in the leaves with β-alanine metabolism, and it is possible that β-alanine metabolism affected carotenoid accumulation (Fig. 6).

Also, the 35 °C RZT treatment increased the concentration of sugars, such as glucose, fructose, and *myo*-inositol, in the leaves, whereas metabolites in the TCA cycle (i.e. fumarate, *cis*-aconitate, and 2-oxoglutarate), G6P and F6P decreased (Fig. 4). This is supported by the previous reports that high temperature-stressed plants accumulate compatible solutes, such as *myo*-inositol, in the cytoplasm to protect the plant from the environmental stress (Yildizli *et al.*, 2018). It is also supported by the fact that plants in stressful conditions accumulated glucose and fructose by an increase in the activity of sucrose-degrading enzymes and a decrease in the activity of hexokinase and fructokinase, which degrade glucose and fructose (Ciereszko *et al.*, 2001; Xue et al., 2008).

Taken together, the 35 °C RZT deteriorated root growth, resulting in a decrease in plant growth and an increase in the content of several pigments (e.g. anthocyanins and carotenoids).

### Altering RZT during plant growth promotes plant growth in the early stages and enhances pigment contents in the later stages

Our first experiment demonstrated that the 25 °C RZT promoted plant growth and that the 35 °C RZT increased pigment contents (Fig. 2). Next, we examined RZT treatments that increased plant biomass and pigment content by combining 25 and 35 °C RZT (Fig. 7). The results showed that the 25 °C RZT treatment resulted in the maximum shoot dry weight (Fig. 8). If maximum shoot yield is the sole and primary objective, then the 25 °C RZT treatment should result in maximum dry shoot yield in these experimental conditions. In contrast, compared with the 25 °C RZT treatment, replacing the 25 °C RZT treatment with 35 °C for 8 days before harvest significantly increased anthocyanins, and replacing the 25 °C RZT treatment with 35 °C for >4 days before harvest decreased leaf dry matter weight (Fig. 8). These results are supported by a study reporting a trade-off between secondary metabolites and plant growth (Yu *et al.*, 2019). Therefore, our study might also have a trade-off between anthocyanins and plant growth in the 35 °C RZT treatment. The present study indicates that changing the RZT from 25 to 35 °C during plant cultivation could promote plant growth in the early stage of plant cultivation at 25 °C RZT while enhancing anthocyanin concentrations in the later stage. It should also be noted that the 35 °C treatment produced a significant decrease in K, As, and Cd in the leaves (Figs 2 and 3), in which high concentrations of K, As, and Cd are considered to be harmful to human health (Chen *et al.*, 2009; Steffensen *et al.*, 2018). In this study, we demonstrated that pre-harvest 35 °C RZT treatment had the potential to accumulate pigment contents (e.g. anthocyanins and carotenoids) and also to reduce harmful substances (e.g. K, As, and Cd). This RZT treatment also potentially leads to the production of increased economic and health-promoting lettuce, although yield productivity is somewhat reduced.

Prior studies have shown that leaf colour is particularly important because it influences consumer purchase behavior and perception of quality (Owen and Lopez, 2015). For example, red leaf lettuce is often used in salad mixes because the red pigment (containing anthocyanins) sells for a higher price than green lettuce (Gazula *et al.*, 2007; Mulabagal *et al.*, 2010). Studies aiming to increase pigment contents in lettuce have generally used ultraviolet or blue light irradiation (Owen and Lopez, 2015; Goto *et al.*, 2016). There are also only a few reports of anthocyanin accumulation induced by reducing the RZT (Sakamoto and Suzuki, 2015; Islam *et al.*, 2019). In the present study, we show, for the first time, that anthocyanins are accumulated by controlling the RZT at an increased temperature. The 25 °C RZT treatment maximizes productivity, and the 35 °C RZT treatment 8 days before harvest allows anthocyanins to accumulate, making it possible to select either high-yielding or high-nutrient-density lettuce in the same number of growing days according to consumer demand. In the future, controlling the environment to accumulate pigment contents is desirable while minimizing the decrease in plant growth. Therefore, whether there is a RZT between 25 and 30 °C that can accumulate pigment contents with less reduction in plant growth is a subject for future research. In addition, it will be necessary to study whether the accumulation of pigment contents can be achieved by combining ultraviolet and blue light irradiation, which have been studied in recent years, and high-temperature control of RZT, while maintaining productivity owing to their synergistic effects.

### Conclusion

To conclude, a red leaf lettuce cultivar was cultivated hydroponically under varying RZTs in order to learn more about its effects on growth and pigment contents. The present study demonstrated successfully that plant growth and pigment contents could be enhanced by adjusting RZT through the cultivation of plants at near-ambient RZT in the early stages and at a high RZT in the later stages. These findings show how control of RZT significantly changes crop characteristics depending on the desired crop characteristics.

More research would be needed to identify the optimal RZTs more accurately, instead of 10 °C increments. In future studies, additional treatments increasing RZT from 25 °C in 1 °C increments can be pursued to find more optimal RZT levels. The methodology of this experiment can be used as a platform for further RZT optimization if desired.

Furthermore, by varying the duration of the RZT treatments and combining ultraviolet and blue light irradiation with RZT control, we could explore further environmental controls that would be optimal for both yield and pigment contents. Ultimately, this could create a new pathway that enables farmers and consumers to customize plant factory food according to supply and demand.

## SUPPLEMENTARY DATA

Supplementary data are available at *Annals of Botany* online and consist of the following.

Figure 1: network analysis by weighted correlation network analysis was used to cluster metabolites and elements in roots that were altered by the RZT treatment. Figure S2: network analysis by weighted correlation network analysis was used to cluster metabolites and elements in leaves that were altered by the RZT treatment. Figure S3: relationship between RZT and dissolved oxygen content (DO).

mcad127_suppl_Supplementary_Figures

## Data Availability

The data that support our paper can be requested by contacting the corresponding author.
