# Supplementary material for: Controlling root zone temperature improves plant growth and pigments in hydroponic lettuce
Source: Ann Bot. 2023 Sep 9;132(3):455–70. doi: 10.1093/aob/mcad127 (PMC10667003; doi:10.1093/aob/mcad127)
Supplement: mcad127_suppl_Supplementary_Figures [file mcad127_suppl_supplementary_figures.pptx]

## Slide 1
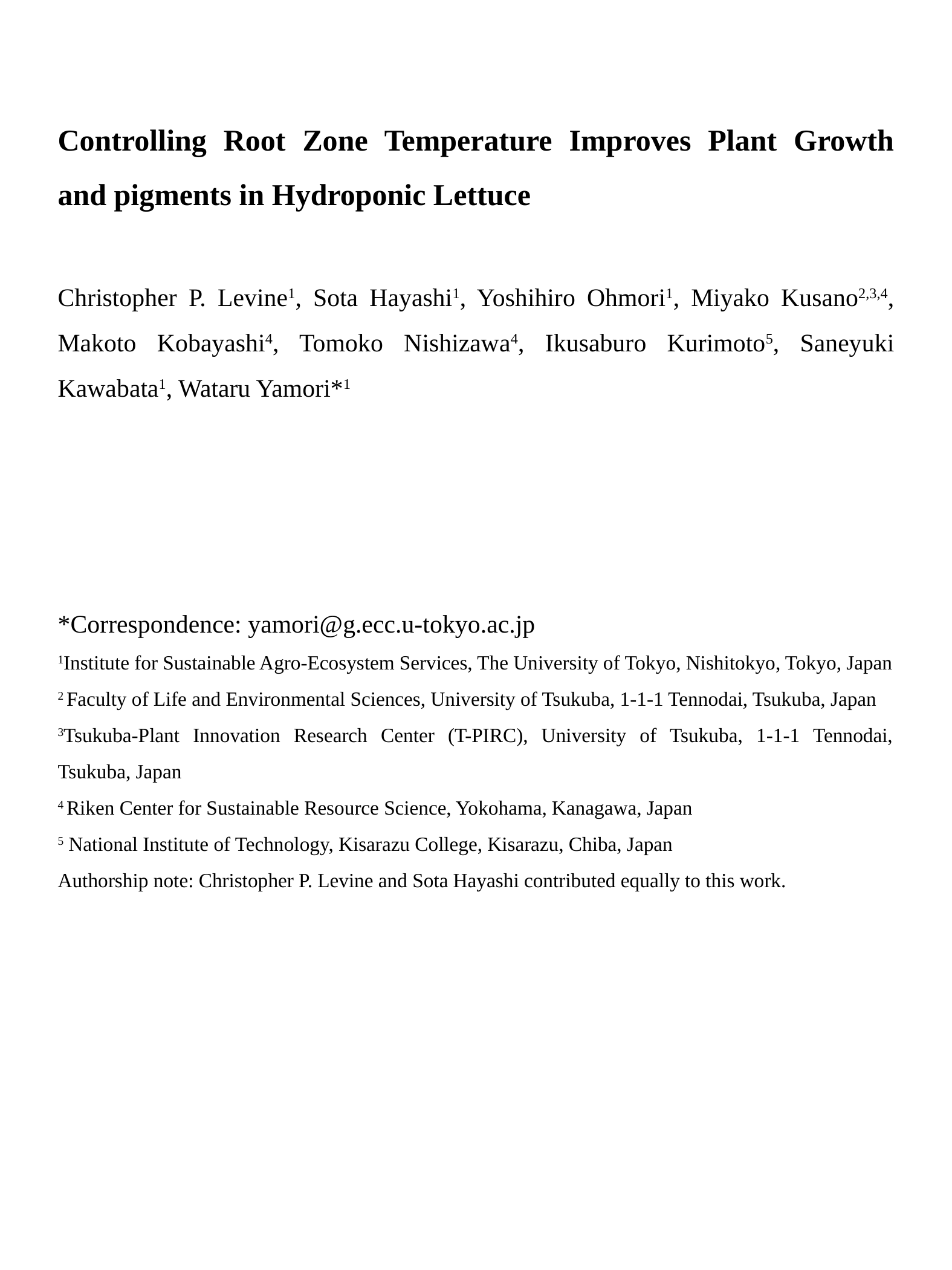

Controlling Root Zone Temperature Improves Plant Growth and pigments in Hydroponic Lettuce
Christopher P. Levine1, Sota Hayashi1, Yoshihiro Ohmori1, Miyako Kusano2,3,4, Makoto Kobayashi4, Tomoko Nishizawa4, Ikusaburo Kurimoto5, Saneyuki Kawabata1, Wataru Yamori*1
*Correspondence: yamori@g.ecc.u-tokyo.ac.jp
1Institute for Sustainable Agro-Ecosystem Services, The University of Tokyo, Nishitokyo, Tokyo, Japan
2 Faculty of Life and Environmental Sciences, University of Tsukuba, 1-1-1 Tennodai, Tsukuba, Japan
3Tsukuba-Plant Innovation Research Center (T-PIRC), University of Tsukuba, 1-1-1 Tennodai, Tsukuba, Japan
4 Riken Center for Sustainable Resource Science, Yokohama, Kanagawa, Japan
5 National Institute of Technology, Kisarazu College, Kisarazu, Chiba, Japan
Authorship note: Christopher P. Levine and Sota Hayashi contributed equally to this work.

## Slide 2
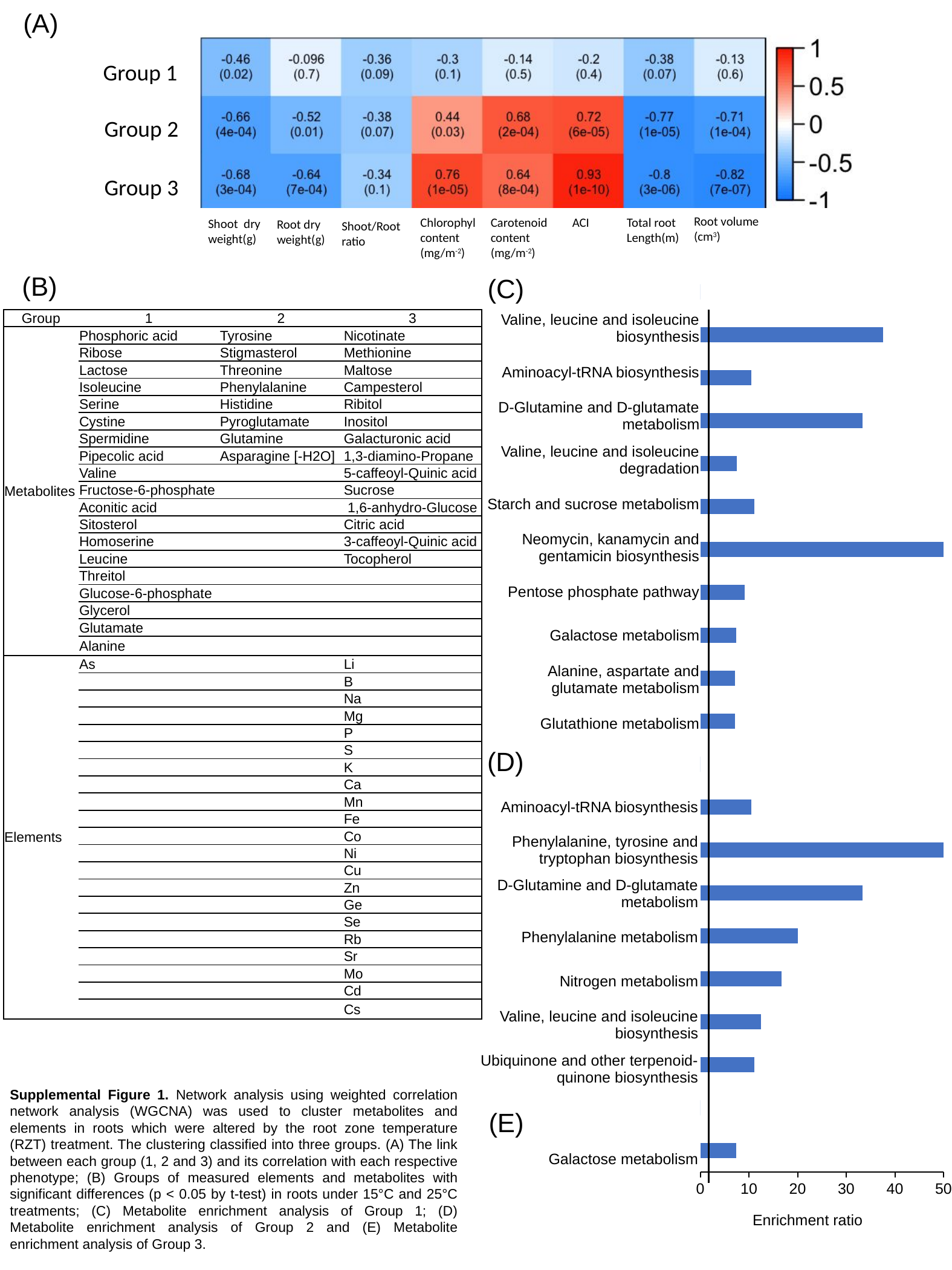

(A)
Group 1
Group 2
Group 3
Root volume
(cm3)
Carotenoid
content
(mg/m-2)
ACI
Total root
Length(m)
Chlorophyl
content
(mg/m-2)
Shoot dry weight(g)
Root dry weight(g)
Shoot/Root
ratio
### Chart
| Category | |
|---|---|
| 8 | 0.0 |
| Valine, leucine and isoleucine biosynthesis | 37.5 |
| Aminoacyl-tRNA biosynthesis | 10.416666666666666 |
| D-Glutamine and D-glutamate metabolismhhh | 33.333333333333336 |
| Valine, leucine and isoleucine degradation | 7.5 |
| Starch and sucrose metabolism | 11.11111111111111 |
| Neomycin, kanamycin and gentamicin biosynthesis | 50.0 |
| Pentose phosphate pathway | 9.090909090909092 |
| Galactose metabolism | 7.407407407407407 |
| Alanine, aspartate and glutamate metabolism | 7.142857142857143 |
| Glutathione metabolism | 7.142857142857143 |
| 9 | 0.0 |
| Aminoacyl-tRNA biosynthesis | 10.416666666666666 |
| Phenylalanine, tyrosine and tryptophan biosynthesis | 50.0 |
| D-Glutamine and D-glutamate metabolism | 33.333333333333336 |
| Phenylalanine metabolism | 20.0 |
| Nitrogen metabolism | 16.666666666666668 |
| Valine, leucine and isoleucine biosynthesis | 12.5 |
| Ubiquinone and other terpenoid-quinone biosynthesis | 11.11111111111111 |
| 10 | 0.0 |
| Galactose metabolism | 7.407407407407407 | (B)
 (C)
| Valine, leucine and isoleucine biosynthesis |
| --- |
| Aminoacyl-tRNA biosynthesis |
| D-Glutamine and D-glutamate metabolism |
| Valine, leucine and isoleucine degradation |
| Starch and sucrose metabolism |
| Neomycin, kanamycin and gentamicin biosynthesis |
| Pentose phosphate pathway |
| Galactose metabolism |
| Alanine, aspartate and glutamate metabolism |
| Glutathione metabolism |
| Group | 1 | 2 | 3 |
| --- | --- | --- | --- |
| Metabolites | Phosphoric acid | Tyrosine | Nicotinate |
| | Ribose | Stigmasterol | Methionine |
| | Lactose | Threonine | Maltose |
| | Isoleucine | Phenylalanine | Campesterol |
| | Serine | Histidine | Ribitol |
| | Cystine | Pyroglutamate | Inositol |
| | Spermidine | Glutamine | Galacturonic acid |
| | Pipecolic acid | Asparagine [-H2O] | 1,3-diamino-Propane |
| | Valine | | 5-caffeoyl-Quinic acid |
| | Fructose-6-phosphate | | Sucrose |
| | Aconitic acid | | 1,6-anhydro-Glucose |
| | Sitosterol | | Citric acid |
| | Homoserine | | 3-caffeoyl-Quinic acid |
| | Leucine | | Tocopherol |
| | Threitol | | |
| | Glucose-6-phosphate | | |
| | Glycerol | | |
| | Glutamate | | |
| | Alanine | | |
| Elements | As | | Li |
| | | | B |
| | | | Na |
| | | | Mg |
| | | | P |
| | | | S |
| | | | K |
| | | | Ca |
| | | | Mn |
| | | | Fe |
| | | | Co |
| | | | Ni |
| | | | Cu |
| | | | Zn |
| | | | Ge |
| | | | Se |
| | | | Rb |
| | | | Sr |
| | | | Mo |
| | | | Cd |
| | | | Cs |
 (D)
| Aminoacyl-tRNA biosynthesis |
| --- |
| Phenylalanine, tyrosine and tryptophan biosynthesis |
| D-Glutamine and D-glutamate metabolism |
| Phenylalanine metabolism |
| Nitrogen metabolism |
| Valine, leucine and isoleucine biosynthesis |
| Ubiquinone and other terpenoid-quinone biosynthesis |
Supplemental Figure 1. Network analysis using weighted correlation network analysis (WGCNA) was used to cluster metabolites and elements in roots which were altered by the root zone temperature (RZT) treatment. The clustering classified into three groups. (A) The link between each group (1, 2 and 3) and its correlation with each respective phenotype; (B) Groups of measured elements and metabolites with significant differences (p < 0.05 by t-test) in roots under 15°C and 25°C treatments; (C) Metabolite enrichment analysis of Group 1; (D) Metabolite enrichment analysis of Group 2 and (E) Metabolite enrichment analysis of Group 3.
 (E)
| Galactose metabolism |
| --- |
Enrichment ratio

## Slide 3
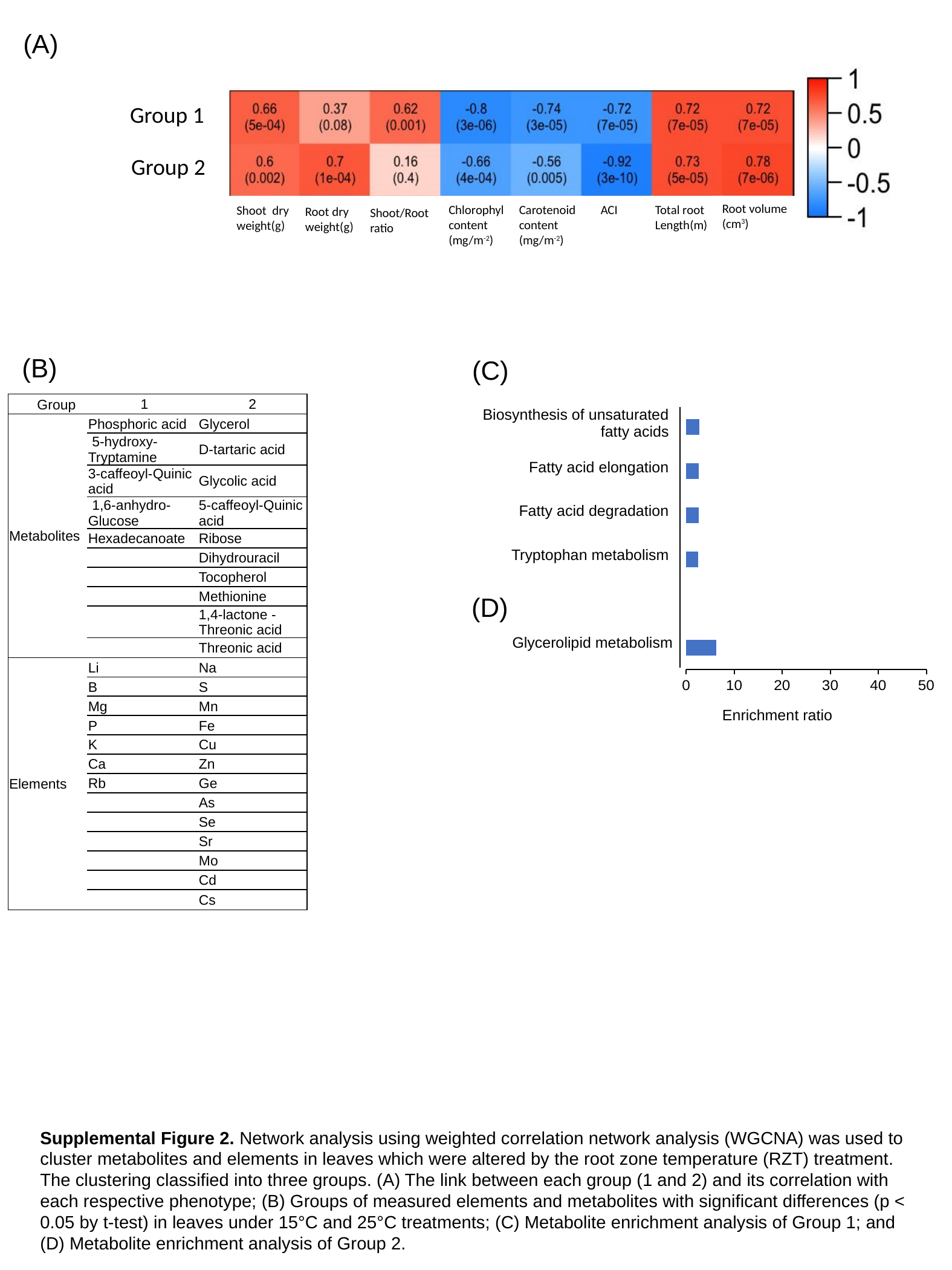

(A)
Group 1
Group 2
Root volume
(cm3)
Carotenoid
content
(mg/m-2)
ACI
Total root
Length(m)
Chlorophyl
content
(mg/m-2)
Shoot dry weight(g)
Root dry weight(g)
Shoot/Root
ratio
 (B)
 (C)
| Group | 1 | 2 |
| --- | --- | --- |
| Metabolites | Phosphoric acid | Glycerol |
| | 5-hydroxy-Tryptamine | D-tartaric acid |
| | 3-caffeoyl-Quinic acid | Glycolic acid |
| | 1,6-anhydro-Glucose | 5-caffeoyl-Quinic acid |
| | Hexadecanoate | Ribose |
| | | Dihydrouracil |
| | | Tocopherol |
| | | Methionine |
| | | 1,4-lactone -Threonic acid |
| | | Threonic acid |
| Elements | Li | Na |
| | B | S |
| | Mg | Mn |
| | P | Fe |
| | K | Cu |
| | Ca | Zn |
| | Rb | Ge |
| | | As |
| | | Se |
| | | Sr |
| | | Mo |
| | | Cd |
| | | Cs |
### Chart
| Category | enrichment ratio |
|---|---|
| Biosynthesis of unsaturated fatty acids | 2.7777777777777777 |
| Fatty acid elongation | 2.6315789473684212 |
| Fatty acid degradation | 2.5641025641025643 |
| Tryptophan metabolism | 2.4390243902439024 |
| 7 | None |
| Glycerolipid metabolism | 6.25 || Biosynthesis of unsaturated fatty acids |
| --- |
| Fatty acid elongation |
| Fatty acid degradation |
| Tryptophan metabolism |
 (D)
| Glycerolipid metabolism |
| --- |
Enrichment ratio
Supplemental Figure 2. Network analysis using weighted correlation network analysis (WGCNA) was used to cluster metabolites and elements in leaves which were altered by the root zone temperature (RZT) treatment. The clustering classified into three groups. (A) The link between each group (1 and 2) and its correlation with each respective phenotype; (B) Groups of measured elements and metabolites with significant differences (p < 0.05 by t-test) in leaves under 15°C and 25°C treatments; (C) Metabolite enrichment analysis of Group 1; and (D) Metabolite enrichment analysis of Group 2.

## Slide 4
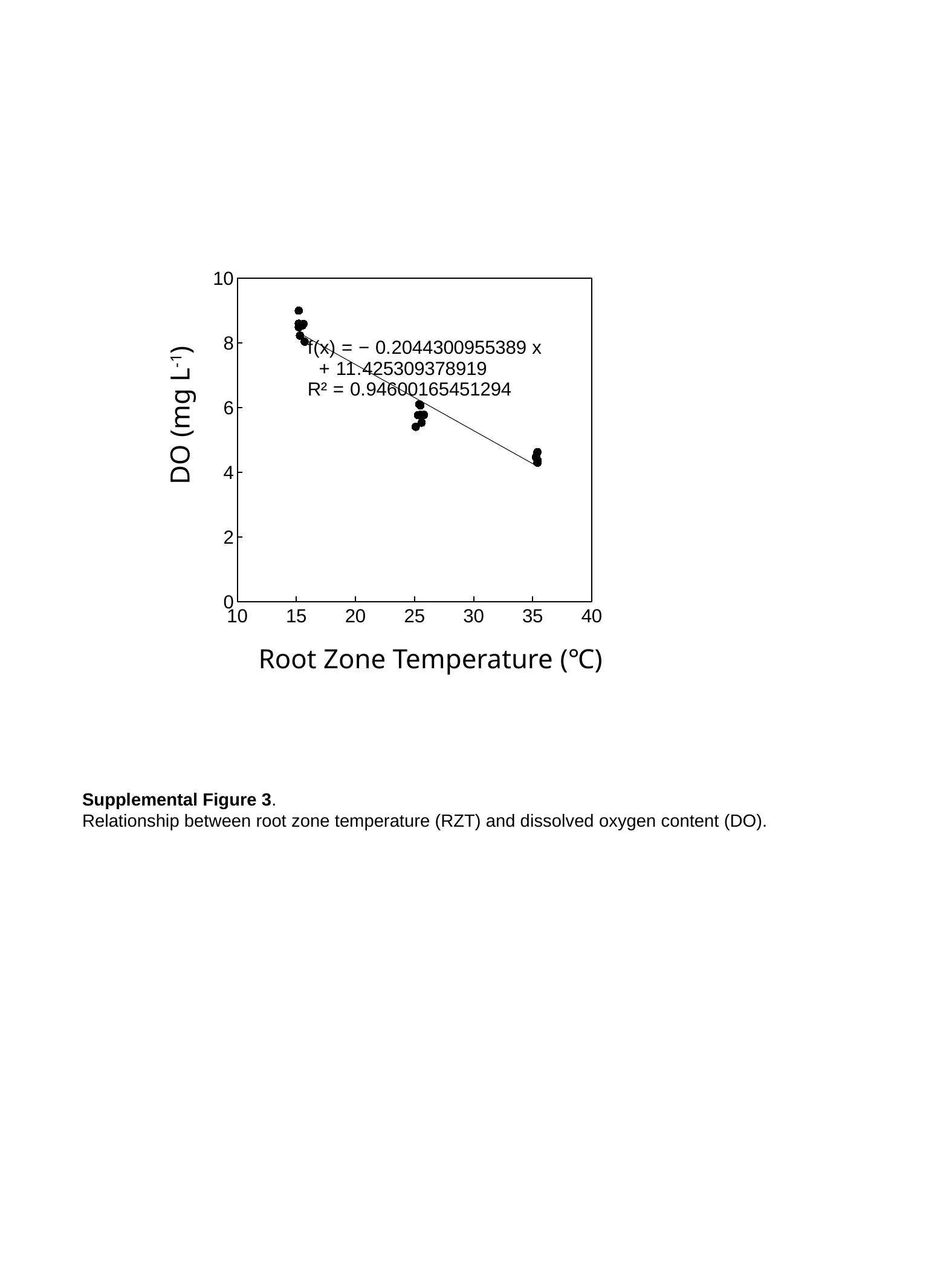

### Chart
| Category | 植物なし |
|---|---|DO (mg L-1)
Root Zone Temperature (℃)
 Supplemental Figure 3.
 Relationship between root zone temperature (RZT) and dissolved oxygen content (DO).
